# Design Parameters of a Miniaturized Piezoelectric Underwater Acoustic Transmitter

**DOI:** 10.3390/s120709098

**Published:** 2012-07-02

**Authors:** Huidong Li, Zhiqun Daniel Deng, Yong Yuan, Thomas J. Carlson

**Affiliations:** Energy and Environment Directorate, Pacific Northwest National Laboratory, P.O. Box 999, Richland, WA 99352, USA; E-Mails: huidong.li@pnnl.gov (H.L.); yong.yuan@pnnl.gov (Y.Y.); thomas.carlson@pnnl.gov (T.J.C.)

**Keywords:** miniaturized transmitter, underwater acoustic transmitter, piezoelectric ceramic, PZT, piezoelectric transducer, transducer power consumption, fish tag

## Abstract

PZT ceramics have been widely used in underwater acoustic transducers. However, literature available discussing the design parameters of a miniaturized PZT-based low-duty-cycle transmitter is very limited. This paper discusses some of the design parameters—the backing material, driving voltage, PZT material type, power consumption and the transducer length of a miniaturized acoustic fish tag using a PZT tube. Four different types of PZT were evaluated with respect to the source level, energy consumption and bandwidth of the transducer. The effect of the tube length on the source level is discussed. The results demonstrate that ultralow-density closed-cell foam is the best backing material for the PZT tube. The Navy Type VI PZTs provide the best source level with relatively low energy consumption and that a low transducer capacitance is preferred for high efficiency. A 35% reduction in the transducer length results in 2 dB decrease in source level.

## 1. Introduction

**Figure 1.**
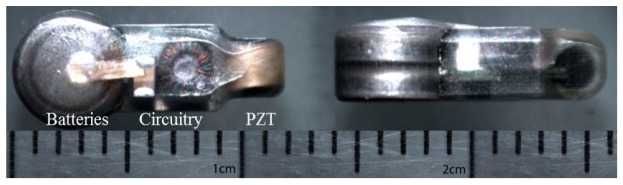
Current JSATS micro transmitters; the top and side views are shown.

The Juvenile Salmon Acoustic Telemetry System (JSATS) is a nonproprietary underwater acoustic sensing technology package that includes piezoelectric acoustic micro transmitters; autonomous, cabled or portable receiving hydrophones; and the corresponding data acquisition/processing software [1–3]. Development of the system was started in 2001 by the U.S. Army Corps of Engineers (USACE), Portland District. The goal was to develop a miniaturized underwater acoustic transmitter small enough to be implanted in juvenile salmon in the Columbia River basin so their migration behavior and survival rates through the Federal Columbia River Power System to the Pacific Ocean could be studied. The latest-generation JSATS micro transmitter is 12 mm in length and weighs 0.43 g in air (Figure 1), small enough for implantation in a 6.5-g fish [3]. However, to study fish species that are even smaller and make the transmitter needle-injectable, a further downsized micro transmitter is needed to lower the probability of mortal injury to the fish [4].

The JSATS micro transmitter consists of three major components—a lead zirconate titanate (PZT) ceramic tube transducer for projecting sound waves, the circuitry that drives the PZT transducer, and the batteries that provide power to the entire transmitter. To further reduce the size and weight of the overall transmitter, possible reductions in the sizes and weights of all three components need to be explored. An effort to reduce the battery weight from its current 270 mg to 100 mg is under way [5]. In the meantime, the JSATS micro transmitter must produce a source level of 156 dB (ref: 1 μPa@1 m) at a frequency of 416.7 kHz to ensure sufficient detection range and efficiency in noisy environments. Thus, it is important that the design for the downsized micro transmitter maintains the required source level.

PZT ceramics have been widely used as the sound-projecting material for various types of acoustic applications, such as fish tags [6], sonar [7], position detectors [8], medical imaging [9], acoustic levitation [10] and cavitation [11], non-destructive testing [12–15] and micro-mixing [16] *etc.* For underwater acoustic applications, most of PZT ceramic transducers reported were much larger in size and focused on lower operating frequencies, compared to what is used in the JSATS. To date, literature reporting comprehensive experimental results for the development of a piezoelectric underwater acoustic transmitter in such a small size is very limited. The objective of this study is to determine some of the key design parameters for the PZT transducer component in the downsized JSATS transmitter, such as the type of the PZT material, the dimensions of the PZT tube, and the type of filler material used inside the PZT tube.

## 2. Experimental Section

### 2.1. Sample Preparation

Generally, for an underwater acoustic projector such as the JSATS micro transmitter, the PZT transducer is preferred to operate at its resonance frequency where the output motion is the greatest so as to produce maximum source level [17]. Due to the heat generation of PZT ceramics during the operation at resonance, hard PZT ceramics with very low loss factor, such as the Navy type I PZTs, are usually used. However, because the duty cycle of the JSATS micro transmitter is extremely low (less than 0.03%) and the size of the transmitter is fairly small, soft PZT ceramics can still be used to produce the optimal source level. To thoroughly investigate the capability of different types of PZT ceramics in the JSATS application, four different types of PZT were evaluated in this study, which include Navy Type I, II, VI, and a modified Type VI PZT, covering a wide range of PZT families from those with “soft” characteristics to those with “hard” characteristics. These PZT ceramic tubes were acquired from EBL Products (East Hartford, CT, USA), Morgan Electro Ceramics (Bedford, OH, USA), and Ferroperm Piezoceramics A/S (Kvistgaard, Denmark), respectively. The inner and outer walls of the PZT tubes were fully coated with electrodes and the tubes were polarized radially through the wall thickness direction. The key piezoelectric and mechanical properties of these materials are listed in Table 1. The dimension range of the PZT chosen for this study was determined by the application frequency of 416.7 kHz.

To measure the source level of different PZT materials with various configurations, electrical leads were first attached to the PZT tubes using a conductive epoxy (CircuitWorks CW2400, ITW Chemtronics, Kennesaw, GA, USA). Appropriate filler materials, which include the CW2400 conductive epoxy, a non-conductive epoxy (301, Epoxy Technology, Billerica, MA, USA), a hot melt glue (ethylene vinyl acetate, FPC Corporation, Wauconda, IL, USA) or EPDM (ethylene-propylene-diene monomer) closed-cell foam (CCF hereinafter, McMaster-Carr, Aurora, OH, USA), were then applied or inserted into the tubes for different transducer configurations. CCF was chosen because of its low density and in consideration of the possible hampering effect that the epoxies may have on the vibration of the PZT tube. For the underwater measurement of the source levels, these tubes were coated with the aforementioned hot melt glue with a thickness of about 0.1 mm, and the electrical leads were soldered onto a coaxial cable to eliminate radio frequency (RF) noise generated from the cable when alternate current (AC) electrical signals were applied to the transducer. All of the epoxies and the hot melt glue were allowed to cure for over 24 h prior to the underwater measurements.

### 2.2. Experimental Setup

**Figure 2.**
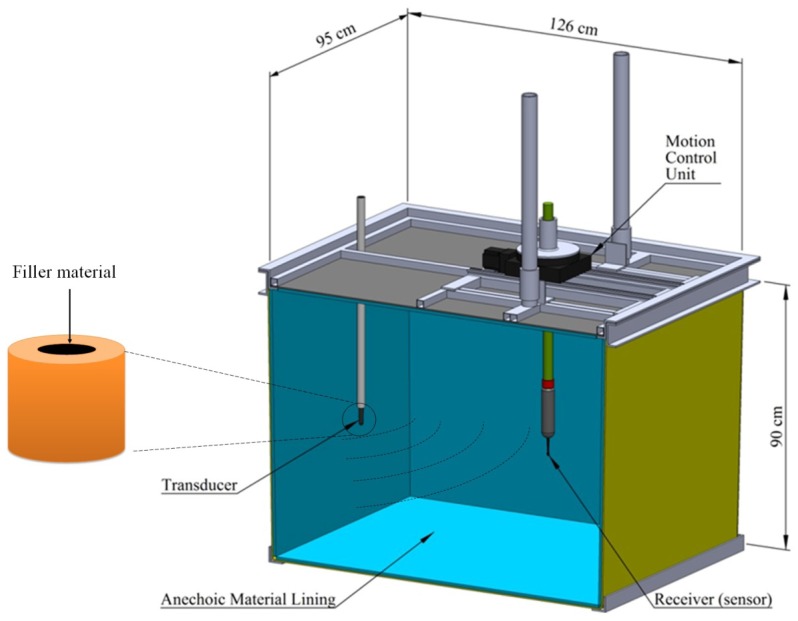
Experimental setup of the source level measurements.

The test setup for the source level measurements consists of an acoustic water tank 1.26 m long, 0.95 m wide, and 0.90 m deep (Figure 2). Inside the tank, all four walls, the tank bottom, and the bottom of the lid were lined with a 26-mm-thick layer of acoustic absorber material (Aptflex F48, Precision Acoustics Ltd., Dorchester, Dorset, UK), which provides excellent ultrasound reflection reduction in the sub-MHz frequency range, thus minimizing the impact of echoes and noises inside the tank during the source level measurement.

To perform the source level measurements, the tank was filled with fresh water. The PZT tube transducer samples fabricated as described above and a receiving hydrophone (Model SC 2008-0004, Sonic Concepts, Bothell, WA, USA) with a 10.6-dB gain were submerged into the tank 17 inches deep. The PZT samples had their tube axis perpendicular to the water surface and were 1 m away from the receiving hydrophone. Both of the samples and the receiving hydrophone were mounted on a motion control unit on top of the acoustic tank so their movement could be controlled three-dimensionally by a Dell Precision T7500 computer workstation (Dell, Round Rock, TX, USA) through a software interface written in MATLAB (MathWorks, Inc., Natick, MA, USA).

Prior to the measurements, the receiving hydrophone was calibrated with an omnidirectional broadband projector hydrophone (Model TC-4034, Reson A/S, Slangerup, Denmark) [18]. An actual JSATS transmitter identification (ID) code was used as the input signal for both calibration and the source level measurements. The ID code uses binary phase shift keying (BPSK) at a frequency of 416.7 kHz and is 31 bits in length, which includes a 7-bit Barker code, 16 data bits, and 8 cyclic redundancy check (CRC) bits. The 31-bit code was sent in a square wave at a pulse rate interval (PRI) of one second from a MATLAB interface through a data acquisition card (Model PCI-6111, National Instruments Corporation, Austin, TX, USA). The source level (SL) was calculated using Equation (1):

(1) $$\text{SL}=20\log_{10}{\text{V}}_{\text{R}}+\text{TL}-{\text{S}}_{\text{R}}-{\text{G}}_{\text{R}}$$

Here, V_R_ is the RMS output voltage of the receiving hydrophone, TL is the transmission loss (in decibels), S_R_ is the sensitivity of the receiving hydrophone, and G_R_ the gain of the data acquisition system.

In the source level measurements for energy consumption characterization, the PZT transducers had the similar sizes (2.54 mm in OD and 1.75 mm in length) and were driven by a JSATS proprietary miniaturized circuitry using an external DC power supply (Tenma 72-6855) at 10 V and 12.5 V. The energy consumptions of the transmitters were obtained by measuring the current draw from the DC power supply during transmission. For other source level measurements, the PZT transducers were driven by an H bridge circuit connected to the DC power supply and a MATLAB interface.

To comprehensively evaluate the source level performance of each PZT material, the PZT transducer was rotated horizontally (tube axis perpendicular to the water surface) through 360° with measurements at 10° intervals while keeping the receiving hydrophone stationary. The source level measurement at each angle was repeated three to five times. In order to be consistent with the testing protocol of the existing JSATS transmitters, the average of all source level measurements between 270° to 90° was used as the representative source level for the PZT transducer, because in the case of a complete JSATS transmitter, the acoustic signal is partially blocked by the circuitry and the battery in the 90°–270° range.

## 3. Results and Discussion

### 3.1. Effect of the Filler Material on Source Level

During operation of the JSATS acoustic micro transmitter, the PZT tube inside the transmitter expands and contracts radially (the “hoop” mode). As a result, sound is radiated from both outer and inner walls of the tube. Because the sound waves radiating from these two surfaces are traveling in opposite directions, sound cancellation becomes a concern. An appropriate filler/backing material inside the PZT tube is thus needed to attenuate the acoustic radiation from the inner surface of the tube [19]. When choosing a backing material, two aspects are usually considered: First, the backing material must have sufficient acoustic attenuation to prevent unwanted reverberations [20]. Second, a backing material with high acoustic impedance might decrease the projecting sensitivity of the transducer [21]. A compromise must thus be made in choosing the appropriate backing material. In the case of JSATS, however, projecting sensitivity (consequently the source level) is more important due to the consideration of sufficient detection range and efficiency in noisy environments.

Four different types of filler materials were applied or inserted into the Morgan PZT508 tubes for this experiment: the Circuitworks CW2400 silver epoxy, the EPO-TEK 301 nonconductive epoxy, hot melt glue, and CCF. The acoustic properties of these materials and the measured source levels are listed in Table 2. The source level of the PZT transducer decreased as the acoustic impedance of the filler material increased. The difference in source level caused by the type of filler material was as large as 7 dB: The source level of the transducer filled with the CW2400 silver epoxy was merely 151.3 dB as a result of heavy damping by the epoxy due to its relatively high acoustic impedance, whereas that of the one with CCF showed 158.5 dB, meeting the source level requirement of the JSATS. This indicates that the very low acoustic impedance of CCF effectively dampens the sound emitted from the inner wall of the PZT tube, ensuring maximum acoustic output from the outer surface of the transducer. Therefore, CCF was chosen as the filler material of the transducer in all the source level results discussed in the subsequent sections of this paper.

### 3.2. Voltage Responses of Different Types of PZT

**Figure 3.**
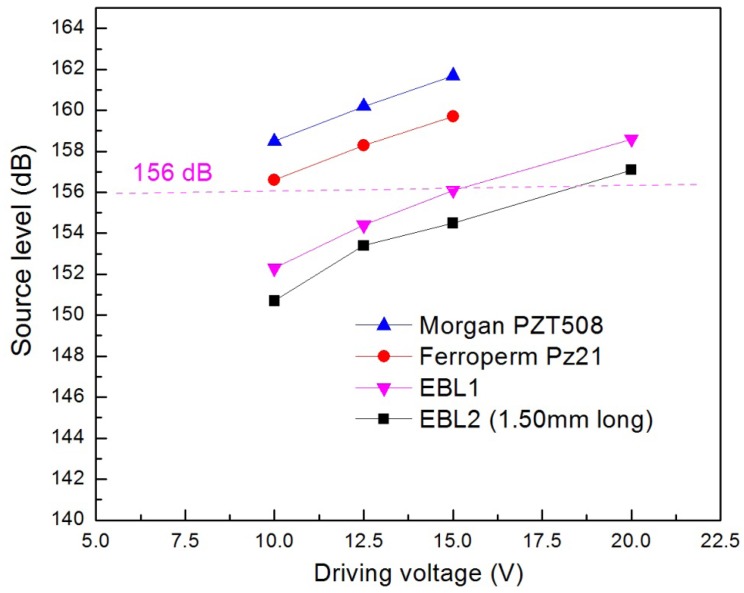
Voltage responses of PZT tubes of different material types (the EBL1 PZT shown in this graph were 1.75 mm long).

Because of the difference in the piezoelectric properties of various types of PZT ceramics, the source levels of the transmitters fabricated using different types of PZT ceramics are expected to vary. To identify the necessary drive voltage to produce the source level of 156 dB (ref: 1 μPa@1 m) that the JSATS required and compare the differences in the projecting ability between different PZT types, the voltage responses of the transducers which were made from the four different types of PZT and had similar sizes were evaluated. The source levels of the transducers scaled with the amplitude of the input voltage in a proportional fashion (Figure 3). At 10 V, both of the very soft PZTs, Morgan PZT508 and Ferroperm Pz21, exhibited excellent source levels above 156 dB, while the hard PZT material, EBL1, showed a source level just about 152 dB. At 20 V, the source level of the transducer made from EBL1 was increased to nearly 159 dB. Therefore, based on the voltage response curve, with the similar dimensions, EBL1, a Type I PZT, required nearly double of the voltage to match the performance of those Type VI PZTs. This was expected, given the fact that it is a harder piezoelectric. Whether this material would provide sufficient benefit in power consumption under the higher drive voltage when compared with soft PZTs is to be investigated after it is incorporated with the circuitry and the battery. EBL2 (1.50 mm long) showed the lowest source levels due to its smallest size.

In addition, the voltage response curves of all the PZT showed fairly similar behavior (Figure 3). With the small size of these PZT transducers, the transducer can be approximated as a spherical source when measuring the sound pressure at 1 m. Theoretically, the far-field acoustic pressure of a small spherical source at distance *r* is expressed by Equation (2) [17]:

(2) $$P(r)=\frac{j\rho {\text{kcaLue}}^{-\text{jkr}}}{2r(1+jka_{s})}$$

where *a* is the mean radius of tube, *L* the length of the tube, *a_s_* the radius of the equivalent sphere $\left(4\pi a_{s}^{2}=2\pi aL\right)$, *j* the square root of −1, *k* the wavenumber, *ρ* the density of water, *c* the speed of sound in water and *u* the normal velocity of the sphere surface. According to Equation (2), the sound pressure is proportional to *u. u* is linearly related to the drive voltage by Equation (3) [17]:

(3) $$u=\frac{2\pi Ld_{31}V}{s_{11}^{E}(j\omega M+2\pi tL/{\text{jas}}_{11}^{E}\omega +R_{m}+Z_{r})}$$

where *a, t, L, M, d_31_* and *s_11_^E^* are the radius, the thickness, the length, the mass, the piezoelectric charge constant, and the elastic compliance of the PZT, respectively; *R_m_* is the mechanical damping resistance, *Z_r_* the radiation impedance and V the driving voltage. Thus, the sound pressure *P* is linearly proportional to *V*. Linear curve fitting of the source level *versus* Log_10_(V), showed a fairly good agreement.

### 3.3. Scaling of Projecting Performances with Energy Consumptions

**Figure 4.**
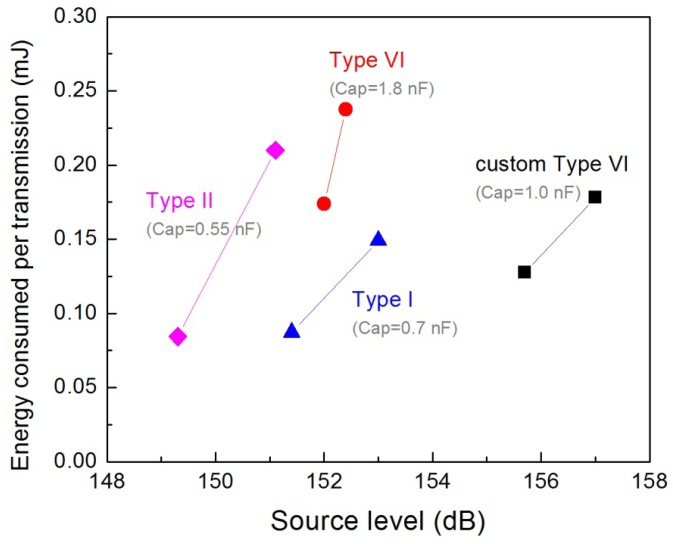
Energy consumption as a function of source level for different types of PZT ceramics.

Generally, for an underwater acoustic projector, the PZT transducer is preferred to operate at its resonance frequency to produce maximum source level [17]. When choosing a piezoelectric material for a near-resonance projector, three important aspects of the transducer need to be considered: the piezoelectric properties, the energy consumption and the heat generated during operation. At a given driving voltage, a soft PZT can provide a higher source level than a hard PZT because of its better piezoelectric properties. However, the lower dielectric constants of the hard PZTs allow for a smaller capacitance of the transducer, which translates into lower power consumption [22]. This is essential for battery-powered micro transmitters. Additionally, the low dielectric loss of the hard PZTs can minimize heat generation during operation. However, if the duty cycle of the transmitter is extremely low and the transmitter size is fairly small, as in the case of JSATS micro transmitters (duty cycle < 0.03%), the soft PZTs can still be used to produce the optimal source level and their slightly higher dielectric loss will not become a concern. But their significantly higher dielectric constants will result in larger transducer capacitance. On the other hand, if a hard PZT is used, the lower piezoelectric properties can be compensated by using a higher driving voltage, which unfortunately also gives rise to higher power consumption. Therefore, in order to determine which PZT type can provide a good balance between source level and power consumption for the JSATS micro transmitter, the source levels and energy consumptions per transmission of the samples made from the four types of PZT were measured at 10 V and 12.5 V. The results including the capacitances of the transducers are shown in Figure 4.

The Type II PZT (EBL2), whose piezoelectric properties lie between those of the Type VI and the Type I PZTs, showed the lowest source level (Figure 4). Although the Type I PZT (EBL1) has the lowest piezoelectric constant and coupling factor, it exhibited higher source levels at lower energy consumption than the Type II PZT. The softest PZT, Morgan PZT508 provided the highest source levels (greater than 155 dB) at relatively low energy consumptions (0.12–0.16 mJ per transmission) although its capacitance (1.00 nF) was higher than those of Type I and Type II transducers (0.70 and 0.50 nF). The energy consumptions of the transducers increased fairly quickly as the drive voltage was increased to enhance the source level. The Pz21, having the largest capacitance among all the samples, was the least efficient: (1) at a similar level of energy consumption, its source level was about 4 dB lower than that of the PZT508 although their piezoelectric properties are largely similar; (2) a steep increase of energy consumption resulted in little gain in source level. Therefore, both piezoelectric properties and transducer capacitance need to be considered for selecting the appropriate piezoelectric material type and dimensions for a miniaturized piezoelectric acoustic projector. For low-duty-cycle applications such as JSATS, Type VI PZTs and small transducer capacitances are preferred.

### 3.4. Frequency Response

**Figure 5.**
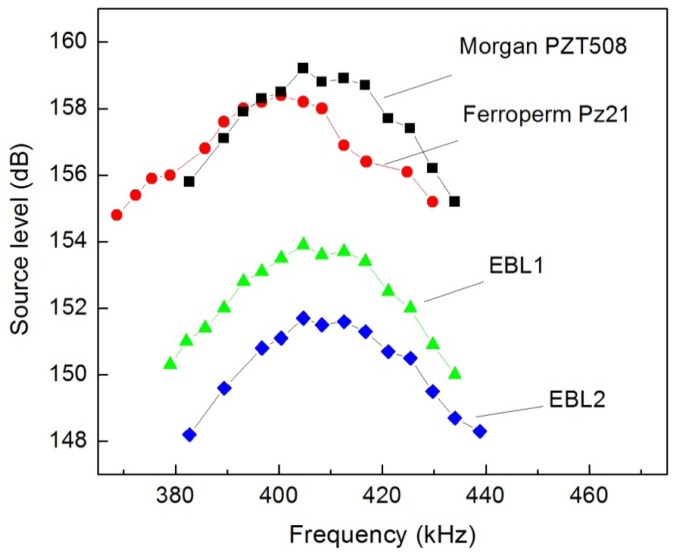
Frequency responses of PZT tubes of different material types.

A piezoelectric acoustic projector is usually preferred to operate at its resonance frequency for maximum energy output. In JSATS, the PZT tube operates in its “hoop” mode at a fixed frequency of 416.7 kHz. The resonance frequency *f_r_* of this mode is determined by Equation (4):

(4) $$f_{r}=\frac{2N}{OD+ID}$$

where *N, OD* and *ID* are the hoop-mode frequency constant, the outer and inner diameters of the tube, respectively. Ideally, the desired OD and ID could be determined using this equation. However, in reality, due to the limitations of the PZT tube manufacturing techniques, some dimensional variations are inevitable. The difference in the mechanical quality factor Q_m_ between PZT types will also cause differences in transducer bandwidths. Therefore, the bandwidths of the transducers need to be investigated to determine the acceptable tolerance range of the resonance required by the application for each PZT type. A similar 1-dB bandwidth of 30 kHz was observed for all four different types of PZT, despite of the differences in their Q_m_s (Figure 5). The CCF backing and the hot-melt glue coating layer used in the transducer fabrication process predominantly damped the transducers, providing a relatively flat frequency response around the resonance, which is a desired characteristic for acoustic projectors [23].

### 3.5. Effect of PZT Tube Length on Source Level

In acoustic transmitter design, the length of the PZT transducer is limited by the size constraint of the transmitter. The length of the PZT tube also affects the source level of the transmitter because it directly relates to the active area radiating the acoustic wave. To study the effect of the tube length on the transmitter source level, EBL1 PZT tubes of five different lengths (2.30, 2.00, 1.75, 1.50 and 1.20 mm) were built and compared.

**Figure 6.**
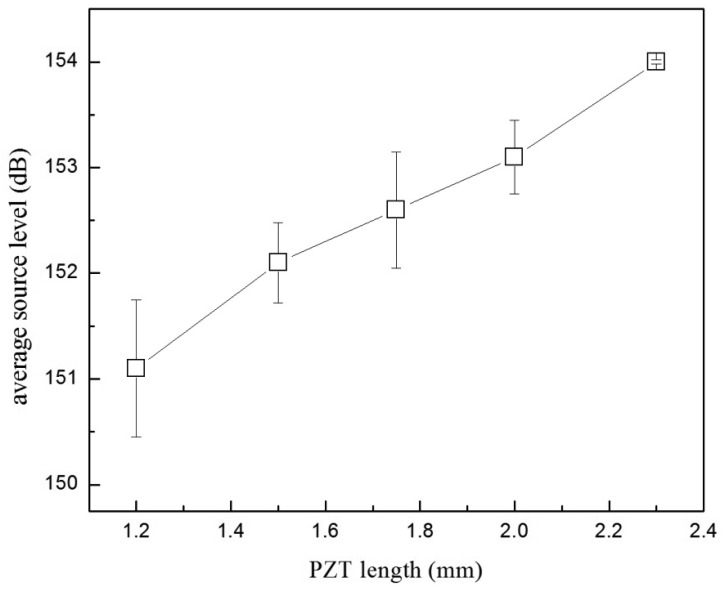
Effect of the PZT tube length on the source level for the EBL1 PZT.

The source level of the 2.30-mm-long PZT tubes was found to be roughly 2 dB higher than that of the 1.50 mm-long ones (Figure 6). This result shows that the reduction in the transducer length (thus the weight) comes with the heavy expense in source level. Source level is a direct reflection of the acoustic energy radiated by the transducer. As the surface of the PZT tube is the active area radiating acoustic signal and all the PZT tubes tested in this experiment had the same OD, the emitted acoustic energy of the transducer should scale linearly with the tube length *L*. A linear fit of the source level *versus* log_10_(*L*) showed a slope value of 9.82, consistent with the theoretical value of 10 [17]. Reduction of source level can be compensated for by an increase in drive voltage, which, however, will consequently impact the battery life. For instance, based on the results in Figure 4, a 2-dB enhancement in source level would require nearly double the energy consumption. Therefore, for a 1.5-mm-long transducer, the capacity of the battery would need to be doubled to achieve similar service life and source level of a 2.3-mm-long transducer. Compromises thus need to be made by weighing the source level, the transducer size and the service life of the transmitter.

## 4. Conclusions

Some of the design parameters—the backing material, driving voltage, PZT material type, power consumption and the transducer length of a miniaturized acoustic fish tag using a PZT tube were evaluated and discussed. The transmitter is used in a low-duty-cycle operation where a high source level and the small size of the transducer are crucial. It was found that closed-cell foam with ultralow density was the best backing material to achieve maximum source level.

As for the selection of the PZT material, the mechanical quality factor of the PZT was shown to have limited influence on the bandwidth of the transmitter as the closed cell foam and hot-melt glue coating of the transducer provided good damping to suppress the large differences in the Q_m_s. In comparison with the soft PZTs, the hard PZTs did not show apparent advantages when comparing the projecting performance at given energy consumptions, although they possessed much lower dielectric constants. Piezoelectric properties of the PZT are shown to have greater impact on the overall performance of the transmitter. The Type VI PZT was determined to be the best material candidate for the JSATS micro transmitter because of the good balance between the source level and power consumption.

A low transducer capacitance is crucial for an efficient battery-powered transmitter. For a cylindrical transducer which operates in the 400 kHz range and requires a source level of 156 dB, as in the case of the JSATS transmitter, a Type VI PZT transducer with an OD of 2.54 mm and a length of 1.65 mm and a driving voltage of 10 V are needed. 1 nF appeared to be a suitable upper limit for the transducer capacitance to achieve good efficiency of the transmitter.

Reducing the PZT length by 35% was found to decrease the source level by 2 dB. The balancing between the transmitter size, service life and acceptable source level has to be considered in the development of the transmitter.

## Figures and Tables

**Table 1.** Dimensions and electrical properties of the PZT ceramic tubes used in this study.

	**Symbol**	**Unit**	**Morgan Electro Ceramics**	**Ferroperm Piezoceramics A/S**	**EBL Products**	
PZT product name			PZT508 (Modified Type VI)	Pz21 (Type VI)	EBL1 (Type I)	EBL2 (Type II)
Outer diameter	OD	mm	2.54	2.54	2.54	2.28
Inner diameter	ID	mm	1.70	1.90	2.10	1.80
Length	L	mm	1.65	1.75	1.20–2.30	1.52
Dielectric constant (@1 kHz)	K_33_^T^		3,900	3,800	1,300	1,725
Dielectric loss (@1 kHz)	tanδ		0.02	0.018	0.004	0.02
Mechanical quality factor	Q_m_		55	65	400	100
Piezoelectric charge constant	d_31_	10^−12^C/N	−315	−250	−127	−173
Coupling coefficient	k_p_		0.71	0.60	0.60	0.62

**Table 2.** Acoustic properties of the filler materials used in this study and the measured source levels.

	**Unit**	**Circuitworks CW2400**	**EPO-TEK 301**	**Hot melt glue**	**EPDM closed-cell foam**
Density	g/cm^3^	2.85	1.15	0.94	0.13
Acoustic impedance	MRayl	Not available	3.05	1.69	Not available
Source level	dB (Ref: 1 μPa@1 m)	151.3	152.5	153.1	158.5
